# A Common Construction Pattern of English Words and Chinese Characters

**DOI:** 10.1371/journal.pone.0074515

**Published:** 2013-09-02

**Authors:** Jiping Huang

**Affiliations:** Department of Physics and State Key Laboratory of Surface Physics, Fudan University, Shanghai, China; University of Maribor, Slovenia

## Abstract

Rankings are ubiquitous around the world. Here I investigate spatial ranking patterns of English Words and Chinese Characters, and reveal a common construction pattern related to phase separation. In detail, I analyze a list of different words in the English language, and find that the frequency of the number of letters per word linearly or nonlinearly decays over its rank in the frequency table. I interpret the linearly decaying area as a linear phase that covers 96.4% words, which is in sharp contrast to a nonlinear phase (representing the nonlinearly decaying area) that covers the remaining 3.6% words. Amazingly, the phase separation phenomenon with the same two percentages of 96.4% and 3.6% holds also for the relation between strokes and characters in the Chinese language although English and Chinese are two distinctly different language systems. The common construction pattern originates from the log-normal distributions of frequencies of words or characters, which can be understood by the joint effect of both the Weber-Fechner law in psychophysics and the principle of maximum entropy in information theory.

## Introduction

The world is full of rankings [1]–[28]: everything, ranging from the reputation of movie stars and academic journals, to purchasing choices, and to a global rich list is affected by differences between them. This turns the quantitative understanding of rankings into a central project of scientific research [1]–[28]. For example, in the English language, the frequency, 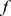, of encountering the 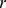
th most common word is inversely proportional to rank order 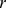 (namely, 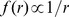), as indicated by Zipf's law [2], [3]. Besides linguistics [2]–[10], Zipfian type power laws (that include Zipf's law [2], [3] and its many extensions [4]–[25] given by 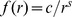 with 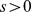, where 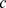 is a non-zero positive constant and 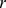 is either rank orders or item's quantities that can be ranked, say, firm sizes [23]) have been observed and studied in many disciplines like physics [18]–[22], acoustics [11], biology [12], [13], economics or finance [15], [16], [24], sociology [17], [23], [25], and architectonics [14]. However, although many rankings can be described by Zipfian type power laws [2]–[25], many others can not, e.g., in communications [26] and linguistics [27], [28]. That is, the ranking patterns might be distinctly different in various areas. Accordingly, in the seminal paper [1], Blumm *et al.* studied temporal ranking patterns of some complex systems. As a result, they revealed a novel noise-driven phase transition that separates stable rankings observed in some complex systems and volatile rankings observed in others. While they made a big success in revealing and explaining the common phase transition phenomenon of different temporal ranking patterns, little academic attention has been devoted to the common phase separation phenomenon of different spatial ranking patterns [that are counterparts of temporal ranking patterns. Here the “temporal ranking pattern” describes the ranking of a specific item (say, the reputation of a particular scientist) that changes with time; the “spatial ranking pattern” depicts the rankings of different items (say, the reputation of a particular scientist versus that of another particular athlete) at a given time]. This is partly because rankings come to appear in such diverse regions that it seems to be an impossible task to obtain a common phase separation behavior. In fact, the failure of existing models of Zipfian type power laws to capture the information-theoretic model for communication [26] also implies the possibility that various spatial ranking patterns share a common phase separation phenomenon. Moreover, understanding the common phase separation phenomenon of different spatial ranking patterns raises some valuable questions: What is the underlying mechanism? Is the mechanism also common for different systems?

To proceed, I attempt to study two different language systems: English and Chinese. Belonging to two different language families, English and Chinese have a lot of significant differences, say, alphabet, phonology, grammar, vocabulary, etc. But do their construction patterns of rankings also differ from each other? To this end, I show that, although the language families are different, their construction patterns of rankings are not: English and Chinese construction patterns of rankings exhibit the same phase separation phenomenon that contains a linear phase that is described by a linearly-decaying law and a nonlinear phase that is described by a nonlinearly-decaying law. Accordingly, the phase separation appears to be “common” for the two different systems, at least to some extent.

## Methods

Let me start by briefly introducing the two languages. The English language contains 54,700 different words [29], each constructed by letters; an individual letter in a word does not represent special meanings (except for 1-letter words like “I”). The Chinese language has 20,893 different characters [30], each constructed by strokes; a stroke in a character has no special meanings either (except for 1-stroke characters like “
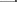
”(one)). In fact, a stroke is only an individual pen movement that is needed to draw a character. For instance, the character “
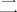
” (two) has two strokes, and the character “
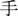
” (hand) has four strokes.

**Figure 1:**
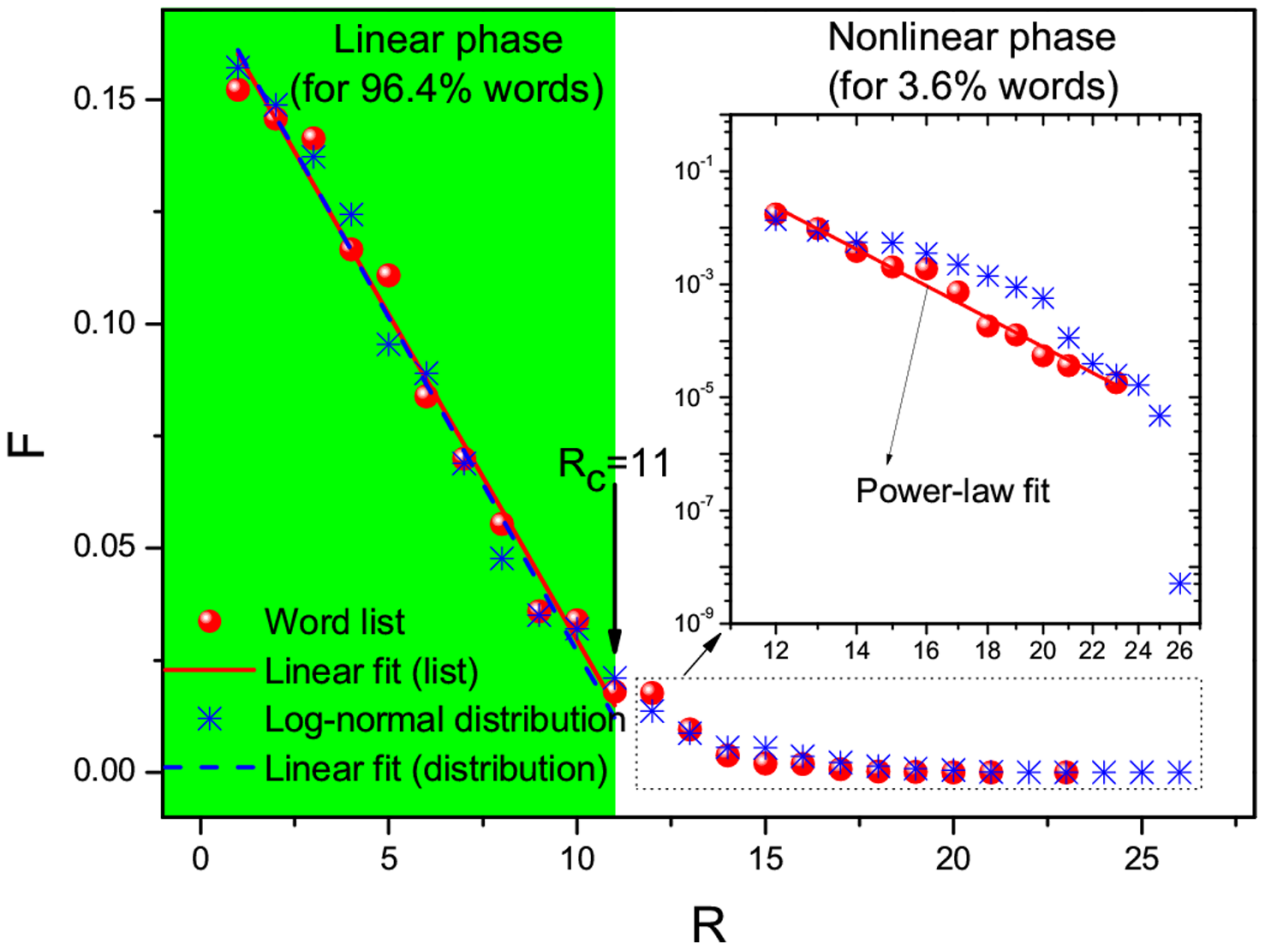
Frequency, *F*, versus rank order, *R*, for the list of 54,700 English words (red circles). For 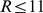, the linear function, 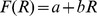, is adopted for the linear fit (red line) covering 96.4% words: 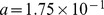 and 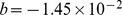. Also shown are the frequencies (blue stars) determined by the log-normal distribution depicted in Fig. 3. For comparison, the same linear function is used to fit the data of frequencies obtained from this log-normal distribution for the same range of 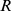: 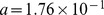 and 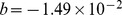 (blue dashes). On the other hand, for 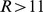 covering the remaining 3.6% words, I attempt to use a power-law distribution function, 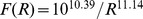 with regression coefficient 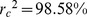 (note the perfect fit corresponds to 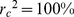 [31]); see the inset that shows a log-log plot. The linear fits are obtained by the least square method. In analogy with critical phenomena, I indicate a critical threshold, 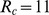. For 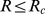, the linearly-decaying behavior described by the linear function comes to appear; I interpret this as a linear phase. For 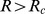, the nonlinearly-decaying behavior occurs, which can be described by nonlinear functions (say, a power-law distribution function as used in the figure); I interpret this as a nonlinear phase.

**Figure 2:**
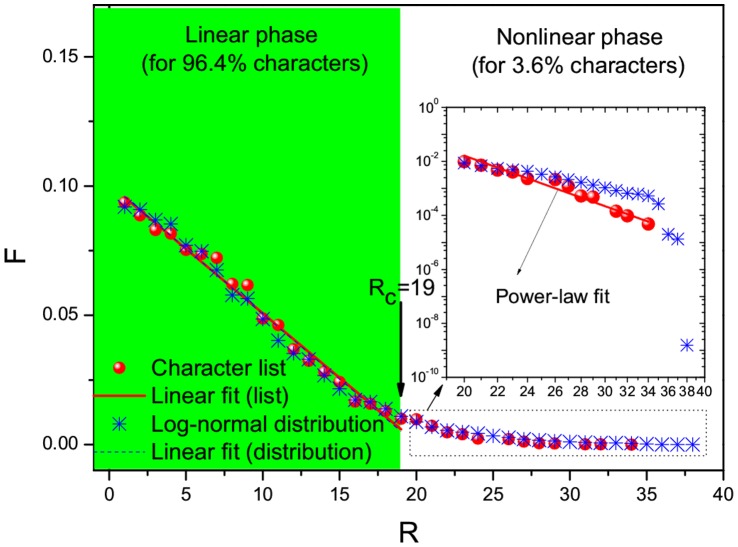
Frequency, *F*, versus rank order, *R*, for the list of 20,893 Chinese characters (red circles). For 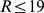, the linear function, 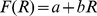, is adopted for the linear fit (red line) covering 96.4% characters: 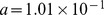 and 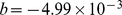. Also shown are the frequencies (blue stars) according to the log-normal distribution depicted in Fig. 4. The same linear function is used to fit the data of frequencies obtained from this log-normal distribution for the same range of 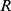: 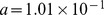 and 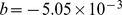 (blue dashes). In addition, for 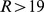 covering the remaining 3.6% characters, I try using a power-law distribution function, 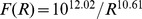 with regression coefficient 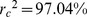; see the inset that depicts a log-log plot. Also, I obtain the linear fits according to the least square method. Following Fig. 1, I also indicate a critical threshold, 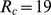, to distinguish the linear phase from the nonlinear phase.

**Figure 3:**
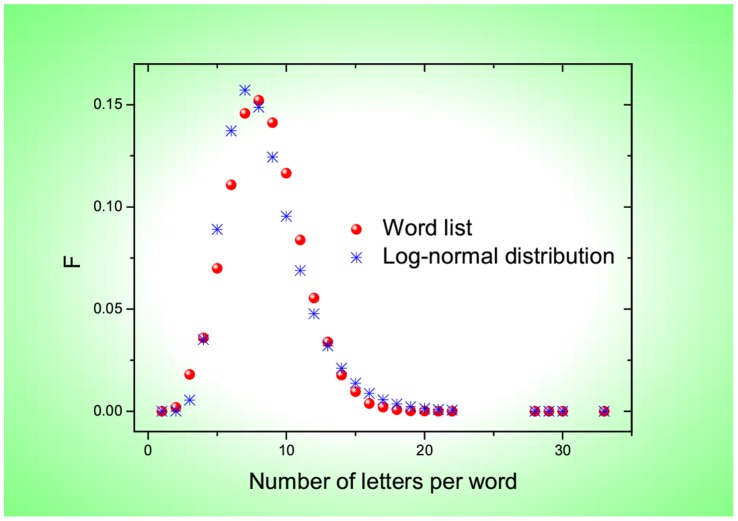
Frequency, *F*, as a function of the number of letters per English word (red circles). Note that the function is approximated with a log-normal distribution (blue stars) that has the same mean value (2.0794) and standard deviation (0.3351) as those determined by the whole list of 54,700 English words.

**Figure 4:**
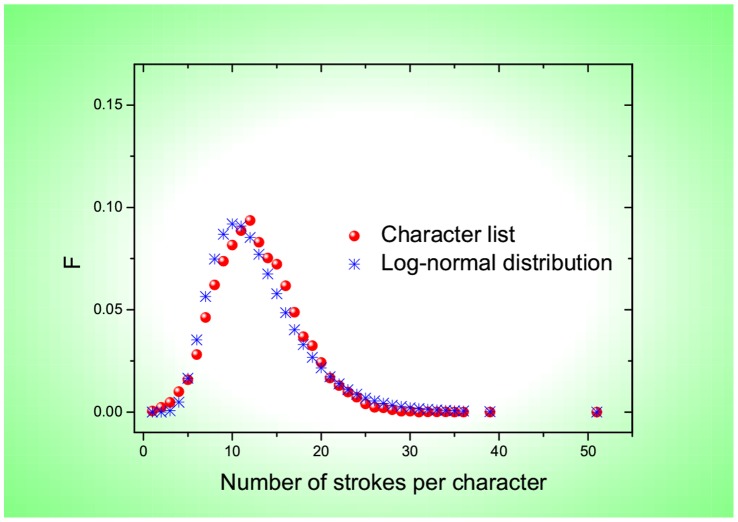
Frequency, *F*, as a function of the number of strokes per Chinese character (red circles). Also, the function is approximated with a log-normal distribution (blue stars) that has the same mean value (2.4835) and standard deviation (0.3896) as those determined by the whole list of 20,893 Chinese characters.

Consider a list of numbers of letters (or strokes) per English word (or per Chinese character), each corresponding to a certain number, 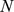, of English words (or Chinese characters) that determines their ranking. I consider that the number of letters or strokes with the largest 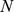 (that is 8326 for English words with 8 letters long and 1957 for Chinese characters with 12 strokes, respectively) is ranked first, namely, its rank order 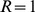. Similarly, I can get 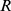 = 2, 3, 4, 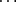. The corresponding data are listed in Table 1.

**Table 1:** 54,700 English words vs 20,893 Chinese characters.

The number		The number		Rank order	
Letters per word	Strokes per character	English words	Chinese characters	English	Chinese
1	1	2	11	21	28
2	2	104	49	16	24
3	3	984	100	11	22
4	4	1967	208	9	19
5	5	3824	331	7	17
6	6	6063	587	5	14
7	7	7972	966	2	11
8	8	8326	1298	1	8
9	9	7729	1540	3	6
10	10	6375	1706	4	4
11	11	4588	1852	6	2
12	12	3028	1957	8	1
13	13	1856	1734	10	3
14	14	969	1573	12	5
15	15	527	1509	13	7
16	16	209	1289	14	9
17	17	111	1018	15	10
18	18	40	771	17	12
19	19	10	678	18	13
20	20	7	506	19	15
21	21	3	348	20	16
22	22	2	271	21	18
28	23	1	204	23	20
29	24	1	153	23	21
30	25	1	84	23	23
33	26	1	49	23	24
	27		44		26
	28		25		27
	29		10		29
	30		10		29
	31		1		34
	32		2		32
	33		3		31
	34		2		32
	35		1		34
	36		1		34
	39		1		34
	51		1		34

On the other hand, I use frequency, 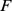, to measure the occupation ratio defined by the quotient of 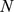 and the total number of English words or Chinese characters.

## Results

As shown in Figs. 1 and 2, most of the frequencies are well approximated by a linearly-decaying law, 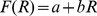. The fact that the linearly-decaying behavior spans 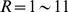 for English words and 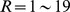 for Chinese characters indicates that the linearly-decaying law is valid for most English words or Chinese characters. Remarkably, the same percentage 96.4% is covered for both English words and Chinese characters. Accordingly, as shown in Figs. 1 and 2, the 96.4% words or characters appear in a linear phase where a linear function works for fitting. In contrast, for the remaining 3.6% words or characters, they are located in a nonlinear phase where a nonlinear function fits instead. Regarding the nonlinear function, what I adopt for Figs. 1 and 2 is power-law distribution functions [31] (based on the private communication with Mr. G. Yang) that belong to the family of Zipfian type power laws. Nevertheless, I should remark that other types of nonlinear functions like exponential distributions might also be suitable due to data sparsity within the current nonlinear phase. Because, compared with the linear phase, the number of words/characters in the nonlinear phase is small enough to be neglected, I would like to focus on the linear phase by raising a question: what is the origin for the observed linearly-decaying behavior? To answer this question, I have to plot the frequency versus the number of letters (or strokes) per English word (or per Chinese character). Figs. 3 and 4 show that the frequencies are approximated with a log-normal distribution for either English words or Chinese characters. This echoes with the findings by Herdan [9] and Zhang [10]. In Ref. [9], Herdan reported that a log-normal distribution appeared for 738 different English words in phone conversations, where the mean value and standard deviation are 5.05 and 1.47, respectively. In Ref. [10], Zhang revealed a log-normal distribution for 16,262 different Chinese characters in the Chinese dictionary “Cihai (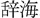)” that was edited as early as 1979, where the mean value and standard deviation are 2.4739 and 0.3827, respectively. When I rank the theoretical values predicted by the two log-normal distributions depicted in Figs. 3 and 4, I find that they agree with those empirically obtained from the 54,700 English words and 20,893 Chinese characters, respectively; see Figs. 1 and 2. Remarkably, they can even be fitted within the same ranges of 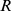 by using the same linear function, 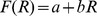, with almost the same parameter sets of 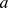 and 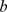. So, I would say the existence of log-normal distributions is a possible origin for the linearly-decaying law.

So far, one may ask why “log-normal distributions” come to appear herein. This can be understood according to the following theoretical analysis (based on the private communication with Dr. J. R. Wei), which is somehow different from the models mentioned in Ref. [32].

Let me set the probability density function of the number (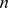) of English words or Chinese characters to be 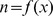. Here 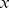 is the number of letters per English word, or the number of strokes per Chinese character. Now I am in a position to introduce both the Weber-Fechner law in psychophysics [33] and the principle of maximum entropy in information theory [34], [35]; see the following two steps.

Step I: According to the Weber-Fechner law, regarding 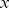, people's psychological perception is 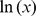. So, for a particular group of people, the distribution of 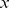 satisfies that the mean and the standard deviation of 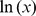 should be constant, respectively.

Step II: The long-time evolution of English words or Chinese characters optimizes 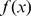. According to the principle of maximum entropy, in order to achieve the optimal 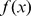, one should maximize information entropy, 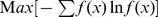.

As a result of the two steps above, one can obtain 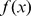 in the log-normal distribution as expected. In other words, this theoretical analysis suggests that the joint effect of both the Weber-Fechner law and the principle of maximum entropy serves as the underlying mechanism for the linear phases shown in Figs. 1 and 2.

## Discussion

The present results indicate that the linearly-decaying law observed in the English and Chinese language systems and the nonlinearly-decaying law observed in the same two systems represent different phases separated by a critical threshold. Despite the difference of the language family of English and Chinese, their spatial ranking patterns can be captured by the same phase separation that displays two distinct phases: Linear phase and nonlinear phase. In analogy with critical phenomena, people might see 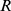 (rank order) as the control parameter and 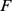 (frequency) as the order parameter. Besides the construction pattern, it is also instructive to compare English and Chinese when they evolve or expand [36], [37].

Uncovering the common phase separation in spatial ranking patterns is expected to have scientific and commercial potentials in some areas with rankings. Below I would like to list some initial thoughts, which might be agendas for future research. First, one may extend the present analysis to other languages or specific books. For books, say, by Shakespeare, one might use this analysis to identify the authenticity of some controversial books. Second, models of rankings [38], [39] are indispensable for models of social organization, ranging from urban or national models to financial market models. The common phase separation might change the conclusions these models offer. In this case, the Bayesian model averaging established by Raftery *et al.* can also help to account for uncertainty about model form [40], [41]. Third, because forecasting ranking patterns is a difficult goal in contrast with the accurate predictive tools common in natural sciences, models describing spatial ranking patterns with a common phase separation become potentially useful for better resource allocation [42], [43] and pricing plans for companies to improve inventory and service allocation. Finally, the common phase separation reported in this work might suggest a class of self-organized critical phenomena, raising the intriguing possibility that besides the usual physical systems like granular piles [44] and proteins [45], the theory of self-organized criticality [46] might also be used to understand ranking systems that people face daily. This also shows that self-organization can serve as an underlying mechanism for not only traditional physical systems, but also non-traditional physical systems [47].
